# Missense and truncating variants in *CHD5* in a dominant neurodevelopmental disorder with intellectual disability, behavioral disturbances, and epilepsy

**DOI:** 10.1007/s00439-021-02283-2

**Published:** 2021-05-04

**Authors:** Ilaria Parenti, Daphné Lehalle, Caroline Nava, Erin Torti, Elsa Leitão, Richard Person, Takeshi Mizuguchi, Naomichi Matsumoto, Mitsuhiro Kato, Kazuyuki Nakamura, Stella A. de Man, Heidi Cope, Vandana Shashi, Undiagnosed Diseases Network, Jennifer Friedman, Pascal Joset, Katharina Steindl, Anita Rauch, Irena Muffels, Peter M. van Hasselt, Florence Petit, Thomas Smol, Gwenaël Le Guyader, Frédéric Bilan, Arthur Sorlin, Antonio Vitobello, Christophe Philippe, Ingrid M. B. H. van de Laar, Marjon A. van Slegtenhorst, Philippe M. Campeau, Ping Yee Billie Au, Mitsuko Nakashima, Hirotomo Saitsu, Tatsuya Yamamoto, Yumiko Nomura, Raymond J. Louie, Michael J. Lyons, Amy Dobson, Astrid S. Plomp, M. Mahdi Motazacker, Frank J. Kaiser, Andrew T. Timberlake, Sabine A. Fuchs, Christel Depienne, Cyril Mignot

**Affiliations:** 1 Institute of Human Genetics, University Hospital Essen, University Duisburg-Essen, Essen, Germany; 2 Département de Génétique, Centre de Référence Déficiences Intellectuelles de Causes Rares, Groupe Hospitalier Pitié-Salpêtrière and Hôpital Trousseau, APHP, Sorbonne Université, Paris, France; 3 Institut du Cerveau (ICM), UMR S 1127, Inserm U1127, CNRS UMR 7225, Sorbonne Université, 75013 Paris, France; 4 GeneDx, Gaithersburg, MD USA; 5 Department of Human Genetics, Yokohama City University Graduate School of Medicine, Yokohama, 236-0004 Japan; 6 Department of Pediatrics, Showa University School of Medicine, Tokyo, 142-8666 Japan; 7 Department of Pediatrics, Yamagata University Faculty of Medicine, Yamagata, 990-9585 Japan; 8 Department of Pediatrics, Amphia Hospital, Breda, The Netherlands; 9 Division of Medical Genetics, Department of Pediatrics, Duke University School of Medicine, Durham, NC 27710 USA; 10 Departments of Neuroscience and Pediatrics, Division of Neurology, Rady Children’s Hospital, UCSD, San Diego and Rady Children’s Institute for Genomic Medicine, San Diego, CA USA; 11 Institute of Medical Genetics, University of Zurich, Schlieren, 8952 Zurich, Switzerland; 12 Rare Disease Initiative Zurich, Clinical Research Priority Program for Rare Diseases University of Zurich, 8032 Zurich, Switzerland; 13 Department of Metabolic Diseases, University Medical Centre Utrecht, Utrecht, The Netherlands; 14 Clinique de Génétique, CHU Lille, 59000 Lille, France; 15 Institut de Génétique Médicale, CHRU Lille, Université de Lille, Lille, France; 16 Service de Génétique Médicale, CHU de Poitiers, Poitiers, France; 17 EA3808 NEUVACOD, University of Poitiers, Poitiers, France; 18 Unité Fonctionnelle d’Innovation Diagnostique des Maladies Rares, FHU-TRANSLAD, France Hospitalo-Universitaire Médecine Translationnelle et Anomalies du Développement (TRANSLAD), Centre Hospitalier Universitaire Dijon Bourgogne, CHU Dijon Bourgogne, Dijon, France; 19 INSERM-Université de Bourgogne UMR1231 GAD « Génétique Des Anomalies du Développement », FHU-TRANSLAD, UFR Des Sciences de Santé, Dijon, France; 20 Centre de Référence Maladies Rares «Anomalies du Développement et Syndromes Malformatifs », Centre de Génétique, FHU‐TRANSLAD, CHU Dijon Bourgogne, Dijon, France; 21 Department of Clinical Genetics, Erasmus MC, University Medical Center Rotterdam, Rotterdam, The Netherlands; 22 CHU Sainte-Justine Research Center, Montreal, QC H3T 1C5 Canada; 23 Sainte-Justine Hospital, University of Montreal, Montreal, QC H3T 1C5 Canada; 24 Department of Medical Genetics and Alberta Children’s Hospital Research Institute, Cumming School of Medicine, University of Calgary, Calgary, AB T2N 4N1 Canada; 25 Department of Biochemistry, Hamamatsu University School of Medicine, Hamamatsu, 431-3192 Japan; 26 Department of Pediatrics, Hirosaki University Graduate School of Medicine and School of Medicine, Hirosaki, 036-8562 Japan; 27 Department of Pediatrics, Hirosaki National Hospital, Hirosaki, 036-8545 Japan; 28 Aomori City Health Center, Aomori, 030-0962 Japan; 29 Greenwood Genetic Center, Greenwood, SC 29646 USA; 30 Department of Clinical Genetics, Amsterdam UMC, University of Amsterdam, Amsterdam, The Netherlands; 31 Laboratory of Genome Diagnostics, Department of Clinical Genetics, Amsterdam UMC, University of Amsterdam, Amsterdam, The Netherlands; 32 Hansjörg Wyss Department of Plastic Surgery, NYU Langone Health, New York, NY USA

## Abstract

Located in the critical 1p36 microdeletion region, the chromodomain helicase DNA-binding protein 5 (*CHD5*) gene encodes a subunit of the nucleosome remodeling and deacetylation (NuRD) complex required for neuronal development. Pathogenic variants in six of nine chromodomain (CHD) genes cause autosomal dominant neurodevelopmental disorders, while *CHD5*-related disorders are still unknown. Thanks to GeneMatcher and international collaborations, we assembled a cohort of 16 unrelated individuals harboring heterozygous *CHD5* variants, all identified by exome sequencing. Twelve patients had de novo* CHD5* variants, including ten missense and two splice site variants. Three familial cases had nonsense or missense variants segregating with speech delay, learning disabilities, and/or craniosynostosis. One patient carried a frameshift variant of unknown inheritance due to unavailability of the father. The most common clinical features included language deficits (81%), behavioral symptoms (69%), intellectual disability (64%), epilepsy (62%), and motor delay (56%). Epilepsy types were variable, with West syndrome observed in three patients, generalized tonic–clonic seizures in two, and other subtypes observed in one individual each. Our findings suggest that, in line with other CHD-related disorders, heterozygous *CHD5* variants are associated with a variable neurodevelopmental syndrome that includes intellectual disability with speech delay, epilepsy, and behavioral problems as main features.

**Supplementary Information:**

The online version contains supplementary material available at 10.1007/s00439-021-02283-2.

## Introduction

The chromodomain-helicase-DNA-binding protein 5 gene (*CHD5*) belongs to a highly conserved family of genes encoding ATP-dependent chromatin remodeling complex subunits comprising nine members, named *CHD1*–*CHD9* (Delmas et al. 1993; Woodage et al. 1997). CHD proteins carry out multiple functions essential for cell survival and embryonic development, including chromatin remodeling, transcriptional regulation, and DNA repair (Tyagi et al. 2016). They are composed of two N-terminal chromodomains important for histone tail binding, a central and conserved SNF2-like helicase motif that uses ATP-hydrolysis for chromatin remodeling, and a less-defined C-terminal DNA-binding domain (Delmas et al. 1993; Woodage et al. 1997). The CHD protein family is further divided into three subfamilies based on the presence or absence of additional domains (Tyagi et al. 2016). Subfamily I (CHD1 and CHD2) features a C-terminal DNA-binding domain that preferentially binds to AT-rich DNA motifs (Tyagi et al. 2016). Subfamily III (CHD6 to CHD9) is characterized by the presence of additional C-terminal functional domains (BRK motif or SANT domain) that define their binding properties (Tyagi et al. 2016). CHD3, CHD4, and CHD5 are part of subfamily II, and, unlike other CHD members, they possess two N-terminal Plant-Homeo Domains (PHD) with histone-binding activity. These three proteins represent mutually exclusive subunits of a large protein complex known as Nucleosome Remodeling and Deacetylase (NuRD) complex (Tyagi et al. 2016).

Subfamily II members are characterized by different expression profiles, with *CHD3* and *CHD4* being ubiquitously expressed, whereas *CHD5* is mainly expressed in brain and testis (Marfella and Imbalzano 2007; Zhuang et al. 2014). Furthermore, the three encoded proteins have distinct, non-redundant properties and functions within the NuRD complex and neuronal defects induced by the specific knockdown of one subunit cannot be rescued by overexpression of another CHD protein (Nitarska et al. 2016). A coordinated sequential switch of these subunits is crucial for mouse cortical development (Nitarska et al. 2016). CHD3 ensures proper layer specification, CHD4 induces early proliferation of the basal progenitors, while CHD5 mediates neuronal differentiation, radial migration, and neuronal cell identity (Nitarska et al. 2016). *CHD5* is required both for activation of genes promoting neuronal differentiation programs and for repression of non-neuronal Polycomb target genes (Egan et al. 2013). Moreover, CHD5 directly interacts with the repressive H3K27me3 histone mark via its PHD and chromodomains (Egan et al. 2013).

All *CHD* genes are evolutionary constrained in human populations, with significantly fewer truncating and missense variants than expected by chance (Karczewski et al. 2020), but only six of the nine *CHD* members have been associated with human disorders so far (Zentner et al. 2010; Merner et al. 2016; Weiss et al. 2016, 2020; Pilarowski et al. 2018; Blok et al. 2018; Chen et al. 2020). Together with *CHD6* and *CHD9, CHD5* has not yet been associated with a human disease. However, *CHD5* is located on chromosome 1p36.31, a region commonly deleted in monosomy 1p36, and *CHD5* haploinsufficiency was hypothesized to contribute to the clinical features of this syndrome, which include neurodevelopmental deficits (intellectual disability with limited language ability), delayed growth, hypotonia, seizures, craniofacial and skeletal features, hearing and vision impairment, as well as cardiac anomalies (Shimada et al. 2015). In this study, we assembled a cohort of 16 unrelated patients with de novo or inherited heterozygous variants in *CHD5*. Comparison of the clinical features of these affected subjects showed that genetic alterations of *CHD5* are associated with a variable neurodevelopmental disorder frequently characterized by intellectual disability (ID), speech delay, motor delay, behavioral problems, and epilepsy.

## Materials and methods

Following the identification by exome sequencing of a de novo missense variant in *CHD5* in a patient with ID, autism spectrum disorder (ASD), and epilepsy, we collected data from additional patients with *CHD5* variants through GeneMatcher (Sobreira et al. 2015). We systematically included all patients with de novo variants as well as patients with either truncating or predicted damaging missense variants inherited from affected parents. Only patients without a detailed clinical history and/or inheritance information were excluded from the study. Exome sequencing was performed at the respective institutions. Referring physicians provided detailed developmental, neurological, and behavioral history of the patients. Patient information was anonymized before data sharing. Variants were described on the *CHD5* NM_015557.3 RefSeq transcript using HGVS recommendations (den Dunnen et al. 2016) and classified according to ACMG guidelines (Richards et al. 2015). All variants have been submitted to the ClinVar Database and have been assigned the following accession numbers: SCV001477999–SCV001478015. Multiple algorithms were used to assess the pathogenicity of *CHD5* variants, including Mutation Taster, Polyphen-2, and SIFT (Ng 2003; Adzhubei et al. 2010; Schwarz et al. 2014). Combined annotation-dependent depletion (CADD) scores (Rentzsch et al. 2019) were calculated for each variant using the GRCh37-v1.6 version (Online Resource Table 1). Prediction of the consequences of the two splicing variants were carried out with Alamut^®^ Visual, a mutation analysis software which includes a splicing module integrating a number of prediction algorithms and splicing prediction data. Nucleotide conservation across 100 vertebrate species was calculated for each variant using the PhastCons score obtained with the phastCons100way.UCSC.hg19 R package (Siepel 2005) and represents the probability that a given nucleotide is conserved (range 0–1). Codon conservation scores were calculated as the mean nucleotide conservation of each triplet. Known *CHD5* NM_015557.3 variants were retrieved from gnomAD v2.1.1 (Karczewski et al. 2020), restricting to loss-of-function, missense, and synonymous single nucleotide variants.

## Results

### *CHD5* variant spectrum

We report 16 different genetic alterations in *CHD5*, including eleven missense variants [c.577C > T, p.(Arg193Trp); c.578G > A, p.(Arg193Gln), c.1279G > A, p.(Glu427Lys); c.2735C > T, p.(Ser912Phe); c.3250G > A, p.(Asp1084Asn); c.3371C > T, p.(Pro1124Leu); c.3407G > A, p.(Arg1136His); c.3419A > T, p.(Asn1140Ile); c.4257C > G, p.(Ile1419Met); c.4463A > T, p.(Asp1488Val) and c.5141A > G, p.(Glu1714Gly)], one duplication of a single base leading to a frameshift [c.612dup, p.(Ser205Leufs*88)], two nonsense substitutions [c.940G > T, p.(Glu314*) and c.1786C > T, p.(Arg596*)], and two splice site variants (c.4079-3C > G and c.4171 + 1G > C). All variants were either absent from gnomAD or present with an allele frequency below 0.0001% (Online Resource Table 1). All missense variants affect highly conserved amino acids of CHD5 (up to zebrafish, Online Resource Fig. 1), had CADD scores above 22, and were predicted to be damaging by at least two algorithms among Polyphen-2, SIFT, and Mutation Taster (Online Resource Table 1). The conservation score of nucleotides and corresponding codons calculated based on the alignment of 100 species additionally indicated that all the affected nucleotides, with the exception of c.4257C > G [resulting in p.(Ile1419Met)], were subject to a great level of conservation during evolution (score 1 in a 0 to 1 scale) (Online Resource Table 1).

**Fig. 1:**
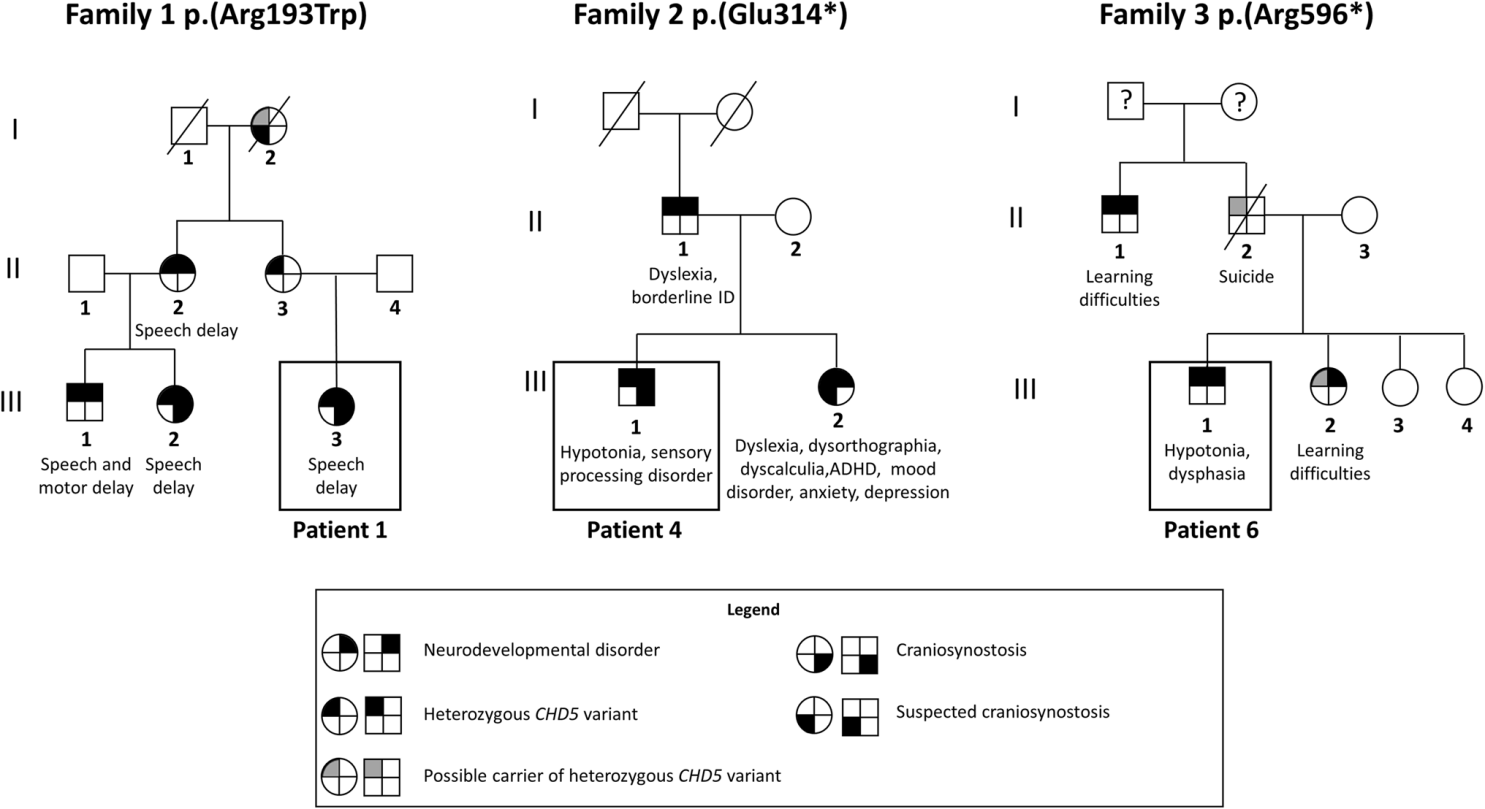
Family trees of the inherited mutations. In family 1, Individual III-3 corresponds to Patient 1. In family 2, Individual III-1 corresponds to Patient 4. In family 3, Individual III-1 corresponds to Patient 6. The variants in *CHD5* identified in these three families are associated with incomplete penetrance and variable expressivity

Ten of the eleven missense substitutions and the two splice site variants occurred de novo in patients without family history, while one missense and the two nonsense variants segregated with neurodevelopmental phenotypes in three families (Fig. 1). The frameshift variant identified in Patient 3 was absent from her mother but inheritance could not be assessed further, since her father was not available for genetic analysis. Notably, one of the missense variants segregating in a larger family [p.(Arg193Trp)] occurred at the same highly conserved residue as one of the de novo missense variants [p.(Arg193Gln)]. Two de novo missense variants [p.(Asn1140Ile) and p.(Ile1419Met)] were mosaic in patients 11 and 14. Both mosaic variants, identified by WES, were present in less than 25% of the total reads on blood DNA and were confirmed by Sanger sequencing.

In addition to these sixteen predicted damaging variants, a de novo variant [c.815C > T, p.(Ala272Val)] absent from gnomAD was identified in a male patient (VUS 1, Online Resource Tables 1 and 2). This variant alters a poorly conserved amino acid located outside of any known domain and is not predicted to alter splicing, but affects a highly conserved nucleotide (score 1) (Online Resource Fig. 1). Because of consistent benign predictions by all algorithms and a CADD score below 20, this variant was considered as of unknown significance despite its de novo occurrence.

### Missense variants in *CHD5* tend to cluster in functional domains

**Fig. 2:**
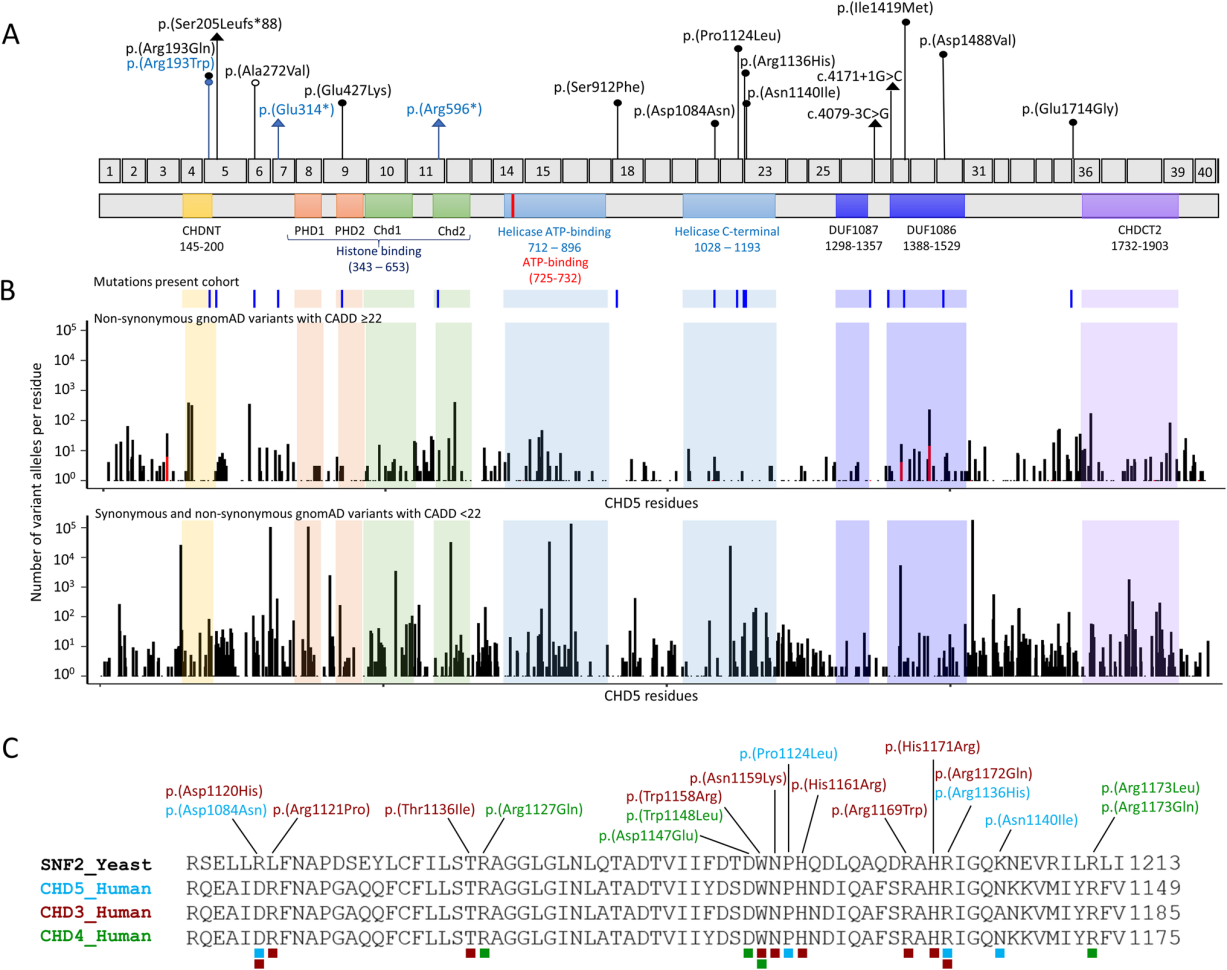
Distribution of the CHD5 variants based on position and conservation of the affected amino acids. **a** Schematic representation of the CHD5 protein and its domains, with position of the identified mutations relative to exon and domain distribution. The CHDNT domain is indicated in yellow, the PHD domains in red, the Chd domains in green, the helicase domains in light blue, the DUFs domains in purple, and the CHDCT2 domain in lilac. Inherited variants are indicated in blue and de novo variants in black. Putative loss-of-function variants are indicated with a triangle, likely pathogenic missense substitutions with a filled circle and the VUS with an empty circle. **b** Comparison of the distribution of the variants identified in our cohort with the synonymous and missense variants reported in gnomAD, with relative position of each affected CHD5 residue across the protein domains. **c** Comparison of a portion of the highly conserved C-terminal Helicase domain among yeast SNF2 (black) and human CHD3 (red), CHD4 (green), and CHD5 (blue). Pathogenic missense substitutions altering residues in this domain are indicated with the color corresponding to the CHD protein where the variant was identified. The amino acids altered by the substitutions are indicated with a square whose color corresponds to the CHD protein where the variant was identified

CHD5 comprises nine protein domains: an N-terminal domain of chromo domain-associated helicases (CHDNT), two PHD domains (PHD1 and PHD2) and two chromodomains (Chd1 and Chd2) important for histone binding, one bipartite Helicase domain with ATPase catalytic activity, two conserved Domains with Unknown Function (DUF1087 and DUF1086), and a C-terminal domain B of chromo domain-associated CHD-like helicases (CHDCT2) mediating the interaction with GATA2D (Pierson et al. 2019) (Fig. 2a). The helicase, PHD, and C-terminal regions are the most conserved and constrained domains (Samocha et al. 2017; Havrilla et al. 2019). Strikingly, missense variants with CADD scores above or equal to 22 reported in gnomAD alter fewer residues in the helicase C-terminal domain (17%) than in other regions (31–42%), contrary to gnomAD synonymous and missense variants with CADD scores below 22, which appeared in 30% of the residues of this part of the helicase domain (other regions 25–60%) (Fig. 2b).

The 11 predicted damaging missense substitutions alter 10 different amino acids and all but two affect a functional domain of CHD5: one variant affects the PHD2 domain, two variants each affect the CHDNT and the DUF1086 domains, and four variants alter the C-terminal part of the helicase domain depleted in missense variants in gnomAD (Fig. 2a, b, Online Resource Fig. 1). Two missense substitutions in the helicase domain match positions altered by two previously published pathogenic variants in *CHD3* (Fig. 2c): CHD5-Asp1084 (Patient 8) corresponds to CHD3-Asp1120, whereas CHD5-Arg1136 (Patient 10) corresponds to CHD3-Arg1172 (Blok et al. 2018). Additionally, Pro1124 (Patient 9) is positioned within a stretch of amino acids that were found to be altered by missense substitutions of *CHD3* (Trp1158, Asn1159, and His1161) or *CHD4* (Asp1147, Trp1148) (Blok et al. 2018; Weiss et al. 2020). Importantly, CHD3 residues Arg1172, Trp1158, and Asn1159 were proven essential for either the ATPase activity of or the ability to carry out chromatin remodeling (Blok et al. 2018).

The nonsense and frameshift variants identified in the present cohort are located in exons 5, 7, and 11, and are therefore predicted to result in transcripts that are subject to nonsense-mediated mRNA decay or to generate a truncated protein, if expressed. The two splice site variants are predicted to, respectively, abolish the acceptor and donor splice sites of exon 27 with high probability (Online Resource Fig. 2a, b). Since variants altering canonical splice sites frequently lead to skipping of the corresponding exon, these two variants possibly induce the same in-frame deletion of the 93 nucleotides of exon 27. Given the preferential expression of *CHD5* in brain and testis, we postulated that the effects of these two variants on *CHD5* splicing could not be assessed. Surprisingly, we were able to amplify *CHD5* transcript from RNA extracted from blood and fibroblasts. A blood sample of Patient 13 could be subsequently obtained, and the resulting analysis showed the in-frame exclusion of exon 27 in the mutant allele, predicted to cause a deletion of 31 amino acids at the protein level [p.(Glu1360_Ser1391delinsGly)] (Online Resource Fig. 2c). *CHD5* splicing in Patient 12 could not be determined due to the impossibility to obtain additional material.

### *CHD5* variants are associated with developmental delay, intellectual disability, behavioral disturbances, epilepsy, and craniosynostosis

**Fig. 3:**
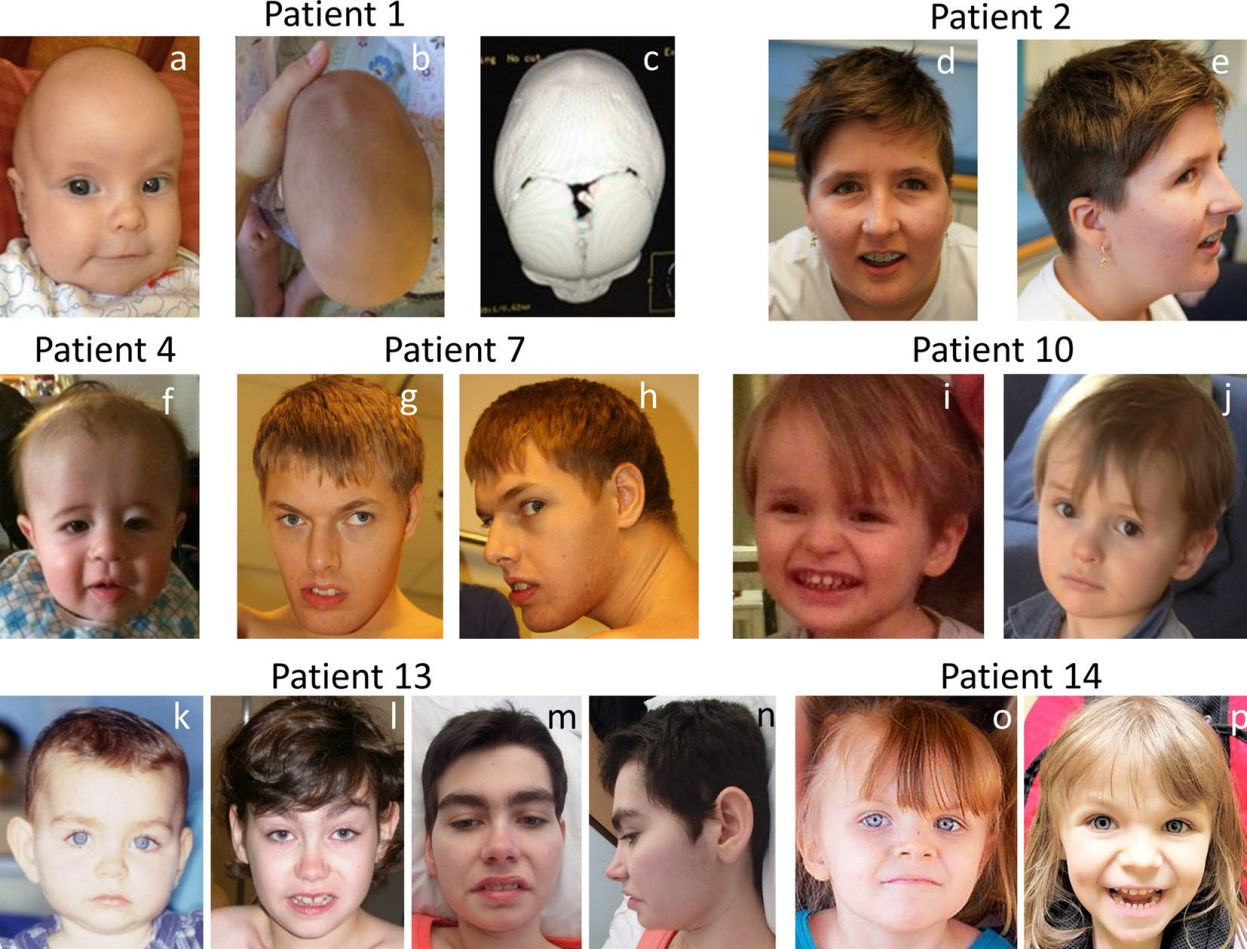
Facial profiles of patients with *CHD5* variants. **a**–**c** Patient 1 age 6 months. **d**, **e** Patient 2 age 11 years 4 months. **f** Patient 4 age 1 year. **g**, **h** Patient 7 age 24 years. **i**, **j** Patient 10 age 3 (**i**) and 5 years (**j**). **k**–**n** Patient 13 age 9 months (**k**), 9 years (**l**) and 22 years (**m**, **n**). **o**, **p** Patient 14 age 3 years six months (**o**) and 6 years (**p**). Facial dysmorphism was related to craniosynostosis in Patients 1 and 4. Other patients displayed subtle facial features, such as high forehead, but no consistent facial dysmorphism emerges from the whole panel

With the exclusion of Patient VUS1, the present cohort comprises seven females and nine males aged from 3 to 24 years (median age 9 years 6 months). Detailed phenotypical information for each patient is provided in Online Resource Table 2. For most patients, pregnancy was unremarkable, birth parameters were normal, and the neonatal period was uneventful. Measurements at the last evaluation were also mainly on average, with only two and four patients presenting with more than two standard deviations above the mean of growth standards for weight and height, respectively. The most frequent clinical features observed in this cohort are summarized in Table 1, and comprise speech delay (*n* = 13/16), behavioral disturbances (*n* = 11/16), epilepsy (*n* = 10/16), subtle facial dysmorphism (*n* = 11/16), motor delay (*n* = 9/16), intellectual disability (*n* = 9/14), hypotonia (*n* = 7/14), and craniosynostosis (*n* = 3/7). The level of intellectual disability could be assessed for six of the nine patients and was moderate in two patients and severe in four. Four patients presented with normal IQ, with a full-scale IQ ranged between 85 and 105, and one patient was reported to have an IQ above average. Developmental milestones were delayed in the majority of the patients, with language acquisition being more affected than motor development. Sitting and walking independently were achieved at a median age of 13 and 28 months, respectively. The first words were pronounced at a median age of 24 months. Three patients were still non-verbal at 3, 9, and 24 years of age. Four patients with an age range between 11 and 22 years could only speak a few words. Dysphasia, stuttering, and echolalia were also reported in single patients. Autism spectrum disorder and obsessive–compulsive tendencies were the most frequently observed behavioral problems in this cohort. Self-injurious behavior, poor eye contact, outbursts of anger, and aggressive behavior were also noted. Seizures occurred in more than half of the patients (*n* = 10) with a median age of onset of 10 months. The earliest onset was at day one and the latest at 16 years of age. Patients could be divided into three groups based on the severity of the seizures, although a significant intra-group variability was also observed: (1) three patients experienced one to five seizures only and were not under antiepileptic therapy; (2) three others had a generalized epilepsy and were still receiving antiepileptic drugs at the time of description; (3) four patients had a diagnosis of developmental and epileptic encephalopathy, and their EEG showed a suppression-burst pattern or hypsarrhythmia. Seizure types included generalized tonic–clonic febrile and afebrile seizures, infantile spasms, generalized staring spells, and myoclonus. Most of the patients were seizure-free at the time of the study with or without specific therapy. Hypotonia was the most frequent finding upon neurological examination (*n* = 7/14), while dysmetria and ataxia were each reported in single patients. Brain Magnetic Resonance Imaging (MRI) were mainly normal (*n* = 8/12) or showed nonspecific abnormalities. Dysmorphic facial features (Fig. 3) were rather nonspecific and did not suggest 1p36 deletion syndrome. They comprised ear anomalies (*n* = 4/16), including low set and posteriorly rotated ears, mildly cupped or smaller ears, prominent nasal bridge and tip (*n* = 3/16), short philtrum (*n* = 3/16), thin upper lip (*n* = 3/16), upslanting palpebral fissures (*n* = 3/16), synophrys (*n* = 2/16), epicanthic folds (*n* = 2/16), frontal bossing (*n* = 2/16), and micrognathia (*n* = 2/16). However, no distinctive facial gestalt emerges from the series. Eye anomalies/visual impairment were noted in some patients but appeared inconsistent within the cohort. Notably, three patients displayed craniosynostosis (Fig. 3). Specifically, Patient 1 displayed sagittal craniosynostosis, Patient 4 had metopic craniosynostosis, and Patient 5 was diagnosed with trigonocephaly. Craniosynostosis of Patients 1 (and of her cousin Individual 1-III-2, see Fig. 1) and of Patient 4 was diagnosed via computed tomography scans by craniofacial surgeons and was surgically corrected by cranial vault remodeling. Patient 5 did not undergo surgery. Malformations of other organs were rare and restricted to individual patients.

**Table 1:**
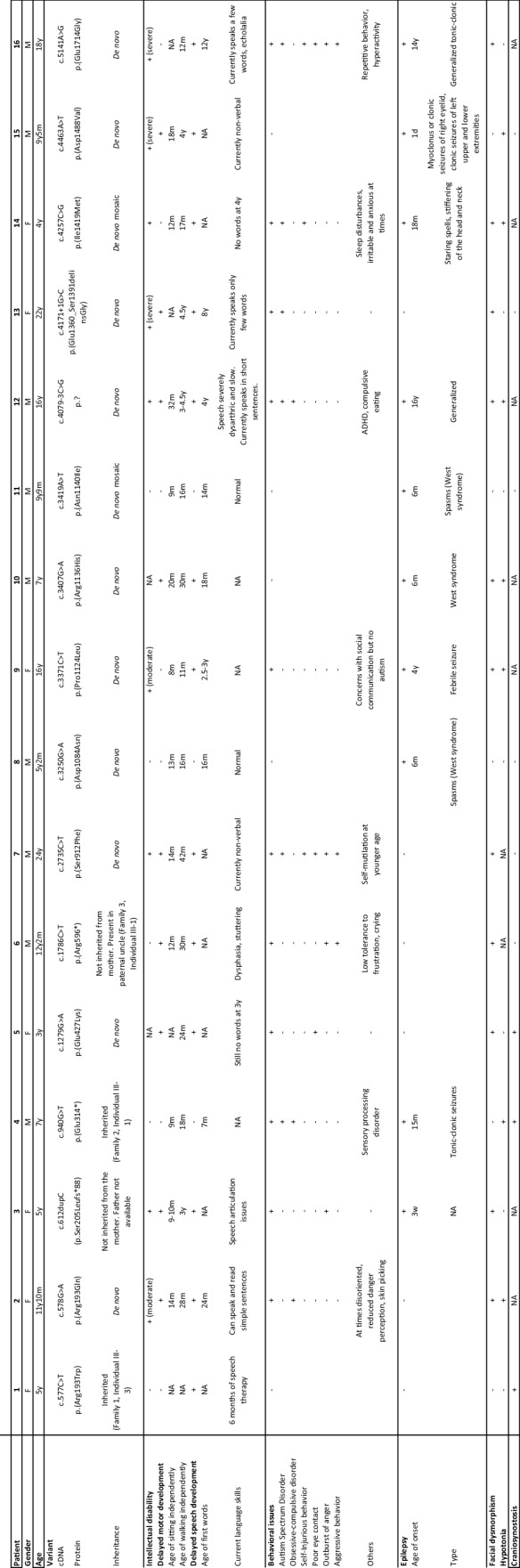
Summary of the main clinical features of patients with CHD5 variants

### Inherited variants are associated with intra-familial variability and incomplete penetrance

Intellectual disability was not diagnosed in any of Patients 1 (family 1), 4 (family 2), and 6 (family 3), and the clinical features were variable within families (Fig. 1). Patient 1, corresponding to individual 1-III-3 (Family 1, Generation III, Individual 3), presented with speech delay, normal motor development, and craniosynostosis. Speech delay was observed also in her cousins 1-III-1 and 1-III-2. Individual 1-III-2 also displayed craniosynostosis, while Individual 1-III-1 was additionally characterized by motor delay and oppositional defiant disorder (detailed clinical data of these two individuals are available in Online Resource Table 2). The p.(Arg193Trp) variant was inherited from the respective mothers, who are sisters. Detailed clinical features were not available for these subjects, but delayed speech was reported for 1-II-2, while her sister 1-II-3 was described as asymptomatic. Suspected craniosynostosis was reported in the maternal grand-mother 1-I-2, who was deceased.

Patient 4 (Individual 2-III-1) had normal development and intellectual abilities, but presented with a sensory processing disorder, obsessive–compulsive tendencies, and was diagnosed with Asperger-like syndrome. He had hypotonia, metopic craniosynostosis, and tonic–clonic seizures over a period of 2 years that spontaneously resolved. His sister (2-III-2) displayed dyslexia, dysorthographia, dyscalculia, mood disorder, anxiety, and depression. The p.(Glu314*) variant present in the two siblings was inherited from their father (2-II-1), who showed dyslexia and borderline intellectual disability.

Patient 6 (3-III-1) presented with mild motor delay and speech impairment, several behavioral problems, mild facial dysmorphism, and strabismus. This family comprises three additional affected members: the proband’s sister (3-III-2) and his paternal uncle (3-II-1) displayed learning difficulties, while his father (3-II-2) had severe psychiatric issues and died from suicide.

Altogether, these data point to the existence of an intra-familial phenotypic variability associated with inherited variants. Moreover, unaffected carriers were also reported in each family, indicating an incomplete penetrance. The lack of a thorough clinical history of each individual could also account for the reported differences.

## Discussion

In this study, we report 13 sporadic cases and 3 families with predicted damaging variants altering highly conserved amino acids of CHD5. Patients with these genetic alterations display a broad spectrum of developmental disturbances, recurrently including developmental delay, learning difficulties or intellectual disability, behavioral problems, seizures, hypotonia, and craniosynostosis. Variants identified in patients include both missense substitutions altering highly conserved amino acids mainly located in functional domains and variants predicted to lead to haploinsufficiency by nonsense-mediated mRNA decay (i.e., frameshift and nonsense variants). The probability that *CHD5* is intolerant to haploinsufficiency, calculated by a recent study including 753,994 individuals, is 0.93 (Collins et al. 2021). *CHD5* is also catalogued among haploinsufficient genes by the Genome Aggregation Database (gnomAD), with a probability to be LOF intolerant (pLI) of 1 and an LOF observed/expected upper bound fraction (LOEUF) of 0.16. Furthermore, its missense *Z*-score (referring to the number of observed and expected missense variants within the same database) indicates that this gene is highly missense-constrained (*Z*-score = 5.32). These metrics indicate that variants disrupting the coding sequence of *CHD5* are counter-selected in human populations, and also suggest that they likely are disease-causing (Karczewski et al. 2020). Nevertheless, 25 out of the 141,456 individuals present in gnomAD harbor *CHD5* variants predicted to be associated with a LOF of the corresponding allele. Incomplete penetrance and variable expressivity, as observed in the familial cases herein reported, could at least partially account for this finding. Furthermore, several of these truncating variants display an allelic imbalance lower than the 0.5 expected for heterozygous variants, suggesting that some of them could be present only at the somatic state in older individuals.

CHD5 is known to play an important role in the context of chromatin remodeling, which it achieves by means of its intrinsic ATPase activity and of its presence within the NuRD complex. Additionally, CHD5 is involved in the regulation of the expression of a subgroup of Polycomb target genes through the maintenance of the repressive H3K27me3 histone methylation mark (Egan et al. 2013). Hence, variants that disrupt CHD5 activity may impact the epigenetic landscape of cells in a way that results in transcriptional disturbances and possibly generates one or several episignatures that are unique for CHD5-related disorders. The pathogenic mechanism(s) by which the variants described in this study contribute to different neurodevelopmental disturbances remains to be defined. Truncating and missense variants could alter the activity of CHD5 and of the NuRD complex in different ways, i.e., by either loss- or gain-of-function, and affect distinct aspects of the epigenetic processes related to the NuRD complex. These mechanisms might include haploinsufficiency of CHD5 within the complex, impaired assembly or composition of the NuRD complex, impaired binding to nucleosomes, or impaired ability to carry out ATP-dependent nucleosome remodeling. The sample size of our cohort and in particular the number of LOF variants was unfortunately too small to establish significant genotype–phenotype correlations based on type and position of the variants. However, from this small cohort, we anticipate that missense substitutions might be more prone to cause epileptic phenotypes. Indeed, approximately half of the patients with missense variants (6/11) had developmental and epileptic encephalopathy and/or an ongoing antiepileptic treatment. In particular, three out of four patients with variants within the helicase domain displayed severe epilepsy (all three patients with West syndrome), while only one patient with variants outside this domain had severe epilepsy (suppression-burst) and two had controlled seizures. Thus, it seems that missense variants, particularly those located in the helicase domain, predispose to early onset epilepsy with a higher likelihood than LOF or missense variants outside this domain. However, this observation needs to be confirmed on larger sample sizes.

*CHD5* is located on chromosome 1p36.31. Patients with *CHD5* variants share nonspecific clinical features with the 1p36 deletion syndrome, a disorder characterized by moderate-to-severe intellectual disability, language deficits, hypotonia, seizures, and distinctive facial features. Depending on the extent of the chromosomal deletion, *CHD5* haploinsufficiency could contribute to the clinical features of this disorder or worsen the severity of intellectual disability, as previously suggested (Shimada et al. 2015). Furthermore, the genes responsible for epilepsy, a frequent feature of the 1p36 deletion syndrome, have not yet been fully characterized. *GABRD* and *KCNAB2* are considered likely candidates for the epileptic phenotype, because patients with a deletion of these genes are more frequently epileptic than those without (Heilstedt et al. 2002; Shimada et al. 2015). However, *CHD5* might also be held accountable for different reasons. With the exception of a single patient (Shimada et al. 2015), *CHD5* was always reported to be deleted together with *KCNAB2*, as *CHD5* is adjacent and proximal to *KCNAB2.* Additionally, point variants in *KCNAB2* have never been described thus far in association with epilepsy. Finally, 16/21 patients with a 1p36 deletion encompassing *CHD5* were reported to display epilepsy, while 19/29 with retained *CHD5* copy do not have epilepsy (Shimada et al. 2015). *RERE* haploinsufficiency might also play a role in the epilepsy of some patients with the 1p36 deletion (Fregeau et al. 2016), but this gene is located proximally to *CHD5* and deleted only in patients with very large deletions [8/50 in (Shimada et al. 2015)]. Thus, *RERE* haploinsufficiency would not explain the epilepsy of most patients with 1p36 deletion. A similar reasoning applies to *SPEN*, a newly identified ID-associated gene with rare seizures, which is proximal to *RERE* (Radio et al. 2021). Taken together, these data suggest that several genes may be involved in the epilepsy phenotype of the 1p36 deletion syndrome and that *CHD5* represents one of its potential modifiers. Notably, seizures are a frequent feature of patients with *CHD5* point variants as well, hence supporting the epileptogenic role of *CHD5* in the context of the 1p36 deletion syndrome.

Patients described in this study also show overlapping features with other neurodevelopmental disorders caused by de novo heterozygous variants in other *CHD* genes, which show intolerance to LOF and missense variants similar to that of *CHD5*. Pathogenic variants in *CHD1* lead to a developmental disorder associated with developmental delay, speech apraxia, autism, hypotonia, and facial dysmorphic features (Pilarowski et al. 2018). *CHD2* pathogenic variants cause a developmental and epileptic encephalopathy (Suls et al. 2013; Chen et al. 2020). Disease-causing variants in *CHD7* and *CHD8* cause CHARGE syndrome and a syndromic form of autism spectrum disorder, respectively (Vissers et al. 2004; Zentner et al. 2010; O’Roak et al. 2011; Merner et al. 2016). Finally, pathogenic variants in *CHD3* and *CHD4* have recently been described in patients with developmental delay, intellectual disability, macrocephaly, impaired speech, and dysmorphic features (Weiss et al. 2016, 2020; Blok et al. 2018; Drivas et al. 2020). Specifically, *CHD3* mutations cause Snijders Blok–Campeau syndrome, which is frequently characterized by autism and signs of connective tissue laxity (Blok et al. 2018; Drivas et al. 2020). *CHD4* mutations cause Sifrim–Hitz–Weiss syndrome, frequently associated with heart malformations as well as numerous other findings (Chiari malformation, Moyamoya disease, hypogonadism, deafness, and limb malformation) (Weiss et al. 2016, 2020). Interestingly, there is an important clinical variability for most *CHD*-related disorders, which makes recognition of these syndromes complicated but yet possible. Notably, seizures are rarely observed in patients with *CHD4* variants and occur only in a minority of patient with *CHD3* alterations. Also craniosynostosis has been rarely reported in association with variants in other *CHD* genes (Siakallis et al. 2019; Tønne et al. 2020). In our cohort, craniosynostosis was observed in two individuals belonging to different families and in one patient with a de novo variant. The specific association of *CHD5* defects with craniosynostosis remains puzzling based on the reported preferential expression of this gene in brain and testis, but possibly suggests that *CHD5* might be expressed more broadly at some stages of embryonic development or that craniosynostosis is linked to an indirect effect of *CHD5* alterations on gene expression programs that coordinate boundary formation or differentiation of overlying cranial neural crest. Interestingly, the knockdown of *chd5*, which shows an expression pattern in adult zebrafish resembling that of *CHD5* in adult human individuals, results in craniofacial development defects including reduced head size and decreased cartilage formation in the head, raising the possibility of additional conserved roles of CHD5 during vertebrate embryogenesis (Bishop et al. 2015). The splicing analysis performed in the present study led to the detection of *CHD5* transcripts also in blood and fibroblasts, suggesting that *CHD5* expression might not be restricted to brain and testis. Hence, a *CHD5* expression pattern that is broader than previously reported could account for the non-brain-related phenotypes observed in this cohort of patients.

*CHD5* is also a known tumor suppressor gene frequently deleted or silenced in diverse human cancers (Bagchi et al. 2007). None of the patients included in this study have had tumors so far, suggesting that germline alterations of *CHD5*, contrary to somatic alterations, do not predispose to a higher risk of tumorigenesis, as previously reported for other tumor suppressor genes, including for instance genes encoding subunits of the SWI–SNF complex or *ASXL1* (Romero and Sanchez-Cespedes 2014; Carlston et al. 2017). However, considering the relatively young age of this cohort, we cannot rule out an increased risk to develop tumors in adult life.

In conclusion, we describe the first cohort of patients with heterozygous variants in *CHD5*, associated with a new syndrome mainly characterized by developmental delay, intellectual disability, behavioral symptoms, and epilepsy. Elaborated functional studies are required to understand the impact of the variants reported in this study on CHD5 protein levels and the NuRD complex during brain development.

## Supplementary Information

Below is the link to the electronic supplementary material.

Supplementary file1 (TIF 3531 kb)

Supplementary file2 (TIF 4553 kb)

Supplementary file3 (XLSX 19 kb)

Supplementary file4 (XLSX 24 kb)

Supplementary file5 (DOCX 16 kb)

## Data Availability

The raw data supporting the results presented in this study are available upon request from the corresponding authors Christel Depienne or Cyril Mignot.
