# Supplementary figures and images for: Missense and truncating variants in CHD5 in a dominant neurodevelopmental disorder with intellectual disability, behavioral disturbances, and epilepsy
Source: Hum Genet. 2021 May 4;140(7):1109–20. doi: 10.1007/s00439-021-02283-2 (PMC8197709; doi:10.1007/s00439-021-02283-2)

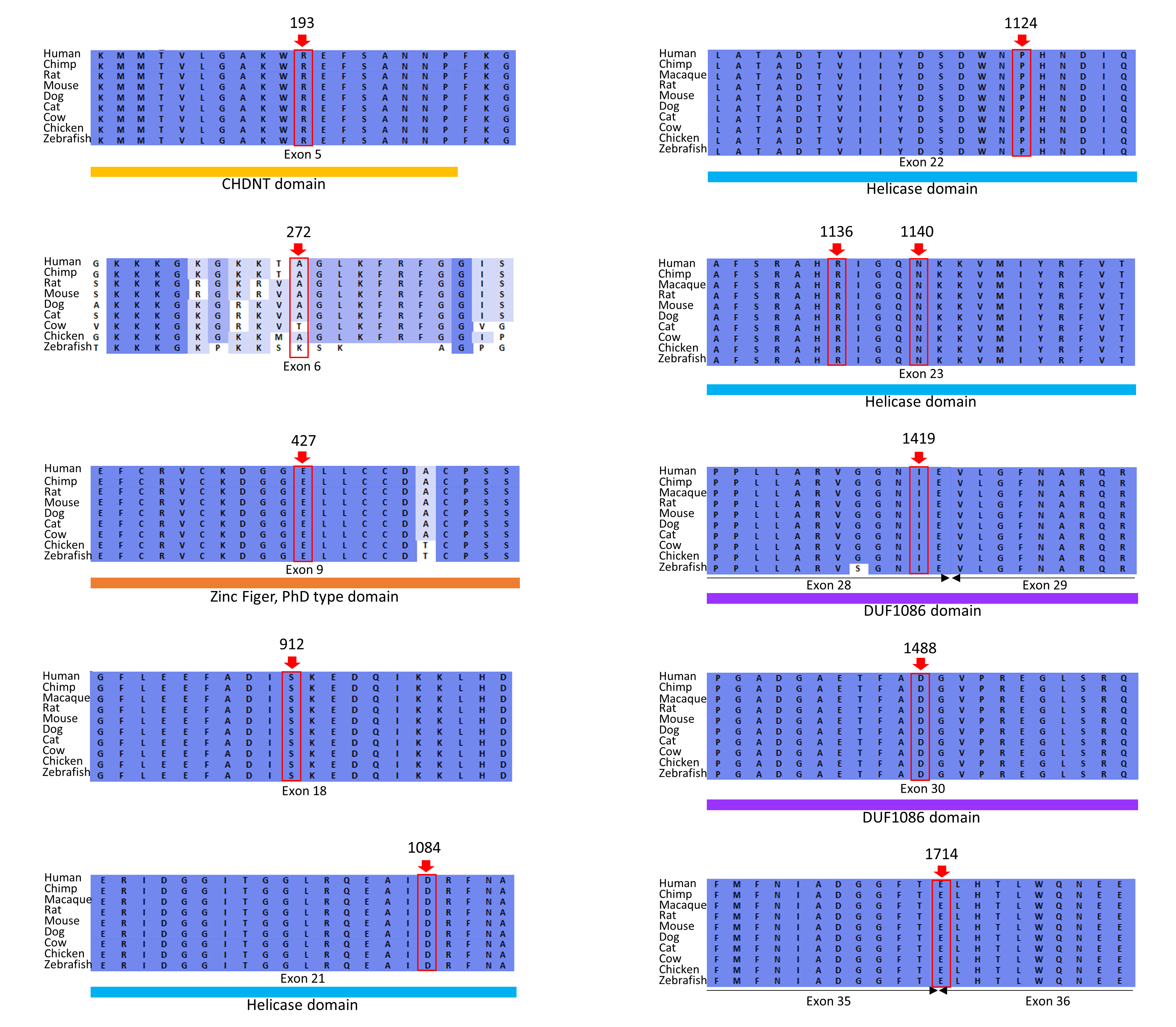

Supplement: Supplementary file 1 — Supplementary file1 (TIF 3531 kb) [file 439_2021_2283_MOESM1_ESM.tif]

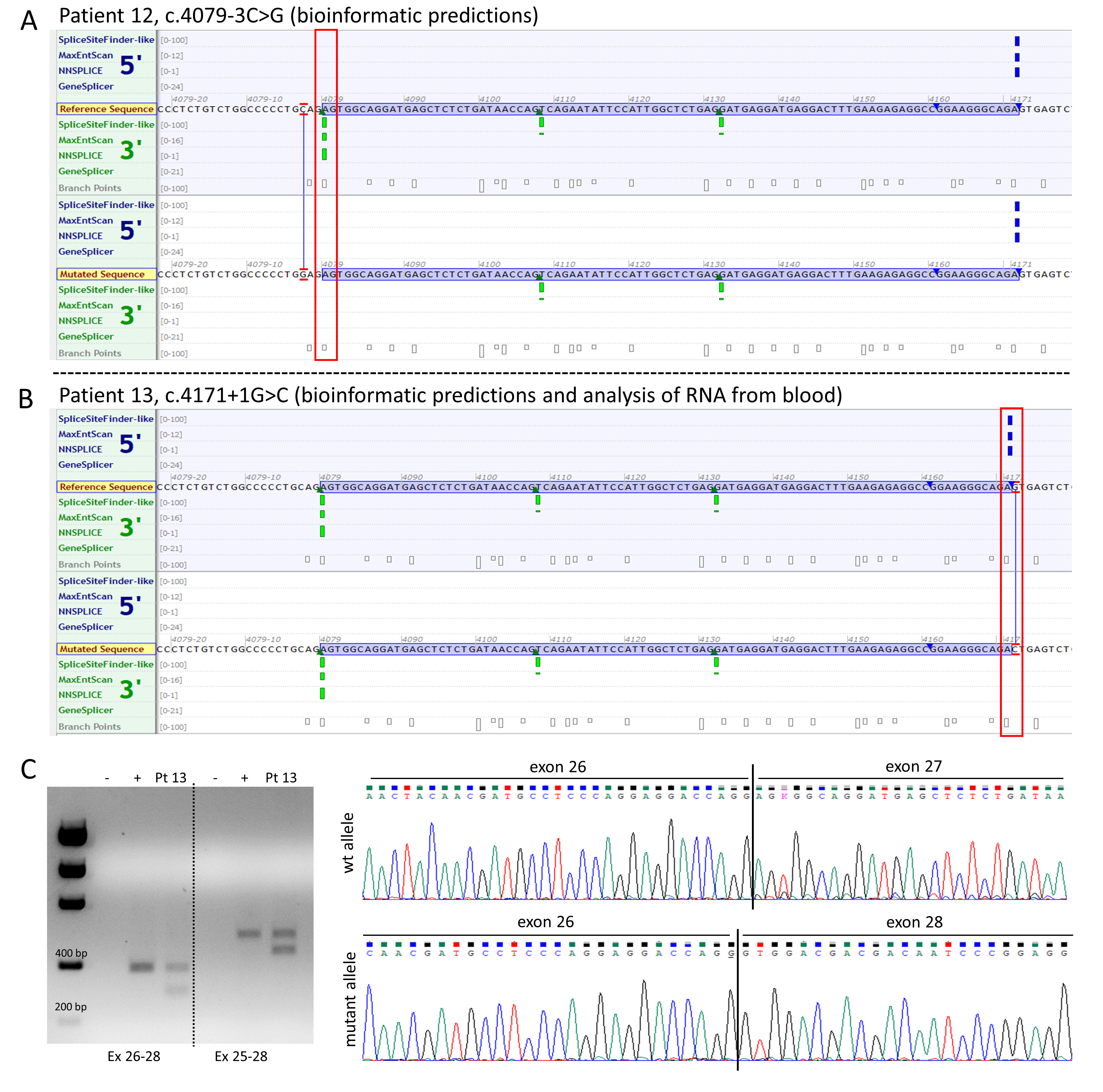

Supplement: Supplementary file 2 — Supplementary file2 (TIF 4553 kb) [file 439_2021_2283_MOESM2_ESM.tif]
