# Supplementary material for: Missense and truncating variants in CHD5 in a dominant neurodevelopmental disorder with intellectual disability, behavioral disturbances, and epilepsy
Source: Hum Genet. 2021 May 4;140(7):1109–20. doi: 10.1007/s00439-021-02283-2 (PMC8197709; doi:10.1007/s00439-021-02283-2)
Supplement: Supplementary file 5 — Supplementary file5 (DOCX 16 kb) [file 439_2021_2283_MOESM5_ESM.docx]

**Online Resource data. List of Undiagnosed Diseases Network consortium members.**

Members of the Undiagnosed Diseases Network include, Maria T. Acosta, Margaret Adam, David R. Adams, Pankaj B. Agrawal, Mercedes E. Alejandro, Justin Alvey, Laura Amendola, Ashley Andrews, Euan A. Ashley, Mahshid S. Azamian, Carlos A. Bacino, Guney Bademci, Eva Baker, Ashok Balasubramanyam, Dustin Baldridge, Jim Bale, Michael Bamshad, Deborah Barbouth, Pinar Bayrak-Toydemir, Anita Beck, Alan H. Beggs, Edward Behrens, Gill Bejerano, Jimmy Bennet, Beverly Berg-Rood, Jonathan A. Bernstein, Gerard T. Berry, Anna Bican, Stephanie Bivona, Elizabeth Blue, John Bohnsack, Carsten Bonnenmann, Devon Bonner, Lorenzo Botto, Brenna Boyd, Lauren C. Briere, Elly Brokamp, Gabrielle Brown, Elizabeth A. Burke, Lindsay C. Burrage, Manish J. Butte, Peter Byers, William E. Byrd, John Carey, Olveen Carrasquillo, Ta Chen Peter Chang, Sirisak Chanprasert, Hsiao-Tuan Chao, Gary D. Clark, Terra R. Coakley, Laurel A. Cobban, Joy D. Cogan, Matthew Coggins, F. Sessions Cole, Heather A. Colley, Cynthia M. Cooper, Heidi Cope*, William J. Craigen, Andrew B. Crouse, Michael Cunningham, Precilla D'Souza, Hongzheng Dai, Surendra Dasari, Joie Davis, Jyoti G. Dayal, Matthew Deardorff, Esteban C. Dell'Angelica, Shweta U. Dhar, Katrina Dipple, Daniel Doherty, Naghmeh Dorrani, Argenia L. Doss, Emilie D. Douine, David D. Draper, Laura Duncan, Dawn Earl, David J. Eckstein, Lisa T. Emrick, Christine M. Eng, Cecilia Esteves, Marni Falk, Liliana Fernandez, Carlos Ferreira, Elizabeth L. Fieg, Laurie C. Findley, Paul G. Fisher, Brent L. Fogel, Irman Forghani, Laure Fresard, William A. Gahl, Ian Glass, Bernadette Gochuico, Rena A. Godfrey, Katie Golden-Grant, Alica M. Goldman, Madison P. Goldrich, David B. Goldstein, Alana Grajewski, Catherine A. Groden, Irma Gutierrez, Sihoun Hahn, Rizwan Hamid, Neil A. Hanchard, Kelly Hassey, Nichole Hayes, Frances High, Anne Hing, Fuki M. Hisama, Ingrid A. Holm, Jason Hom, Martha Horike-Pyne, Alden Huang, Yong Huang, Laryssa Huryn, Rosario Isasi, Fariha Jamal, Gail P. Jarvik, Jeffrey Jarvik, Suman Jayadev, Lefkothea Karaviti, Jennifer Kennedy, Dana Kiley, Isaac S. Kohane, Jennefer N. Kohler, Deborah Krakow, Donna M. Krasnewich, Elijah Kravets, Susan Korrick, Mary Koziura, Joel B. Krier, Seema R. Lalani, Byron Lam, Christina Lam, Grace L. LaMoure, Brendan C. Lanpher, Ian R. Lanza, Lea Latham, Kimberly LeBlanc, Brendan H. Lee, Hane Lee, Roy Levitt, Richard A. Lewis, Sharyn A. Lincoln, Pengfei Liu, Xue Zhong Liu, Nicola Longo, Sandra K. Loo, Joseph Loscalzo, Richard L. Maas, John MacDowall, Ellen F. Macnamara, Calum A. MacRae, Valerie V. Maduro, Marta M. Majcherska, Bryan C. Mak, May Christine V. Malicdan, Laura A. Mamounas, Teri A. Manolio, Rong Mao, Kenneth Maravilla, Thomas C. Markello, Ronit Marom, Gabor Marth, Beth A. Martin, Martin G. Martin, Julian A. Martínez-Agosto, Shruti Marwaha, Jacob McCauley, Allyn McConkie-Rosell, Colleen E. McCormack, Alexa T. McCray, Elisabeth McGee, Heather Mefford, J. Lawrence Merritt, Matthew Might, Ghayda Mirzaa, Eva Morava, Paolo M. Moretti, Deborah Mosbrook-Davis, John J. Mulvihill, David R. Murdock, Anna Nagy, Mariko Nakano-Okuno, Avi Nath, Stan F. Nelson, John H. Newman, Sarah K. Nicholas, Deborah Nickerson, Shirley Nieves-Rodriguez, Donna Novacic, Devin Oglesbee, James P. Orengo, Laura Pace, Stephen Pak, J. Carl Pallais, Christina GS. Palmer, Jeanette C. Papp, Neil H. Parker, John A. Phillips III, Jennifer E. Posey, Lorraine Potocki, Bradley Power, Barbara N. Pusey, Aaron Quinlan, Wendy Raskind, Archana N. Raja, Deepak A. Rao, Genecee Renteria, Chloe M. Reuter, Lynette Rives, Amy K. Robertson, Lance H. Rodan, Jill A. Rosenfeld, Natalie Rosenwasser, Francis Rossignol, Maura Ruzhnikov, Ralph Sacco, Jacinda B. Sampson, Susan L. Samson, Mario Saporta, C. Ron Scott, Judy Schaechter, Timothy Schedl, Kelly Schoch, Daryl A. Scott, Vandana Shashi*, Jimann Shin, Rebecca Signer, Edwin K. Silverman, Janet S. Sinsheimer, Kathy Sisco, Edward C. Smith, Kevin S. Smith, Emily Solem, Lilianna Solnica-Krezel, Ben Solomon, Rebecca C. Spillmann, Joan M. Stoler, Jennifer A. Sullivan, Kathleen Sullivan, Angela Sun, Shirley Sutton, David A. Sweetser, Virginia Sybert, Holly K. Tabor, Amelia L. M. Tan, Queenie K.-G. Tan, Mustafa Tekin, Fred Telischi, Willa Thorson, Audrey Thurm, Cynthia J. Tifft, Camilo Toro, Alyssa A. Tran, Brianna M. Tucker, Tiina K. Urv, Adeline Vanderver, Matt Velinder, Dave Viskochil, Tiphanie P. Vogel, Colleen E. Wahl, Stephanie Wallace, Nicole M. Walley, Chris A. Walsh, Melissa Walker, Jennifer Wambach, Jijun Wan, Lee-kai Wang, Michael F. Wangler, Patricia A. Ward, Daniel Wegner, Mark Wener, Tara Wenger, Katherine Wesseling Perry, Monte Westerfield, Matthew T. Wheeler, Jordan Whitlock, Lynne A. Wolfe, Jeremy D. Woods, Shinya Yamamoto, John Yang, Muhammad Yousef, Diane B. Zastrow, Wadih Zein, Chunli Zhao, Stephan Zuchner

*Authors meeting authorship criteria
